# Investigating the frequency of free-living amoeba in water resources with emphasis on *Acanthamoeba* in Bandar Abbas city, Hormozgan province, Iran in 2019–2020

**DOI:** 10.1186/s13104-020-05267-z

**Published:** 2020-09-05

**Authors:** Homa Attariani, Habibollah Turki, Saeed Shoja, Abdoreza Salahi-Moghaddam, Amin Ghanbarnejad, Jebreil Shamseddin

**Affiliations:** 1 Molecular Medicine Research Center, Hormozgan Health Institute, Hormozgan University of Medical Sciences, Bandar Abbas, Iran; 2 Infectious and Tropical Diseases Research Center, Hormozgan Health Institute, Hormozgan University of Medical Sciences, Bandar Abbas, Iran; 3 Department of Public Health, Social Determinants in Health Promotion Research Center, Hormozgan Health Institute, Hormozgan University of Medical Sciences, Bandar Abbas, Iran

**Keywords:** Free-living amoebae, *Acanthamoeba*, Water resources

## Abstract

**Objective:**

These amoebas can cause dangerous illnesses when they accidentally enter the human body, so it is necessary to determine various forms of organisms in water resources to prevent the danger they can cause and risks to human health. Currently, in Bandar Abbas, there is no sufficient information about the distribution of *Acanthamoeba,* and we intended to study its frequency and determine the related genotypes.

**Results:**

Out of 83 water samples collected from different resources in the city, 31 plates (37.3%) were found to be positive for free-living amoebae. Of these, five were identified as *Acanthamoeba* (6%) by culture method and 8 (9.6%) by molecular method. Positive sample sequence analysis enabled us to distinguish two genotypes of T4 (7 cases) and T15 (1 case) in this study.

## Introduction

Pathogenic and opportunistic free-living amoebae such as *Acanthamoeba* spp., *Balamuthia mandrillaris,* and *Naegleria fowleri* are aerobic, mitochondriate, eukaryotic organisms that occur worldwide and can potentially cause infections in humans and other animals. They are ubiquitous in soil and water resources, aquatic environments, ponds, hot springs, swimming pools, domestic sewage, air, air conditioning chambers, sediments, stagnant water, and artificial humanmade creatures [1]. These amoebae use bacteria, yeasts, and other organisms as food. Unlike real parasites, pathogenic free-living amoebae can complete their life cycle in the environment without entering the human and animal host. An increasing number of people with immunodeficiency diseases, including AIDS, treating the patients with corticosteroids or people undergoing chemotherapy, are at increased risk of developing amoebic infections [2, 3].

In Turkey, environmental samples (100%), in USA 2454 tap water samples (51%), water supply in Osaka, Japan (19%), and in 40 water and sanitation facilities samples in Tunisia Hospital (47.6%) *Acanthamoeba* was found [4–6].

In Iran, some researches covered large areas of Iran to detect various free-living amoeba [7], and some others identified other parasites accompanied by amoeba [8, 9].

*Acanthamoeba* detection is usually carried out based on the structural characteristics of the cyst through direct microscopic diagnostic methods and culture techniques. This technique has its limitations due to the impacts of cultivation conditions. In recent years, the molecular method has largely solved the problem, and the molecular tests are useful confirmation tools for *Acanthamoeba* differentiation from other free-living amoebae [10].

A review on the published papers and articles revealed that no such study had been conducted in Bandar Abbas Hormozgan, Iran, so far. Finally, the objective of this study was to determine the frequency of free-living amoeba in Bandar Abbas water resources by cultivation and polymerase chain reaction (PCR) methods.

## Main text

### Methods

Bandar Abbas city is the capital of Hormozgan province. The city is located in the north of the Hormuz Strait, which is located on the shores of the Persian Gulf. The city encompasses about a total area of 45 km^2^, and its height above the sea level is 10 m. Hormozgan province is one of the hot and dry regions of Iran, and its climate is affected by semi-desert and desert climate. The climate of the coastal strip is sweltering and humid in summers, and sometimes its temperature exceeds 52 ºC. The average annual temperature in this area is about 27 ºC. Hormozgan province's climate is characterized by a long warm season and a temperate short season. The warm and humid weather lasts 9 months [11].

#### Samples and sampling sites

**Fig. 1:**
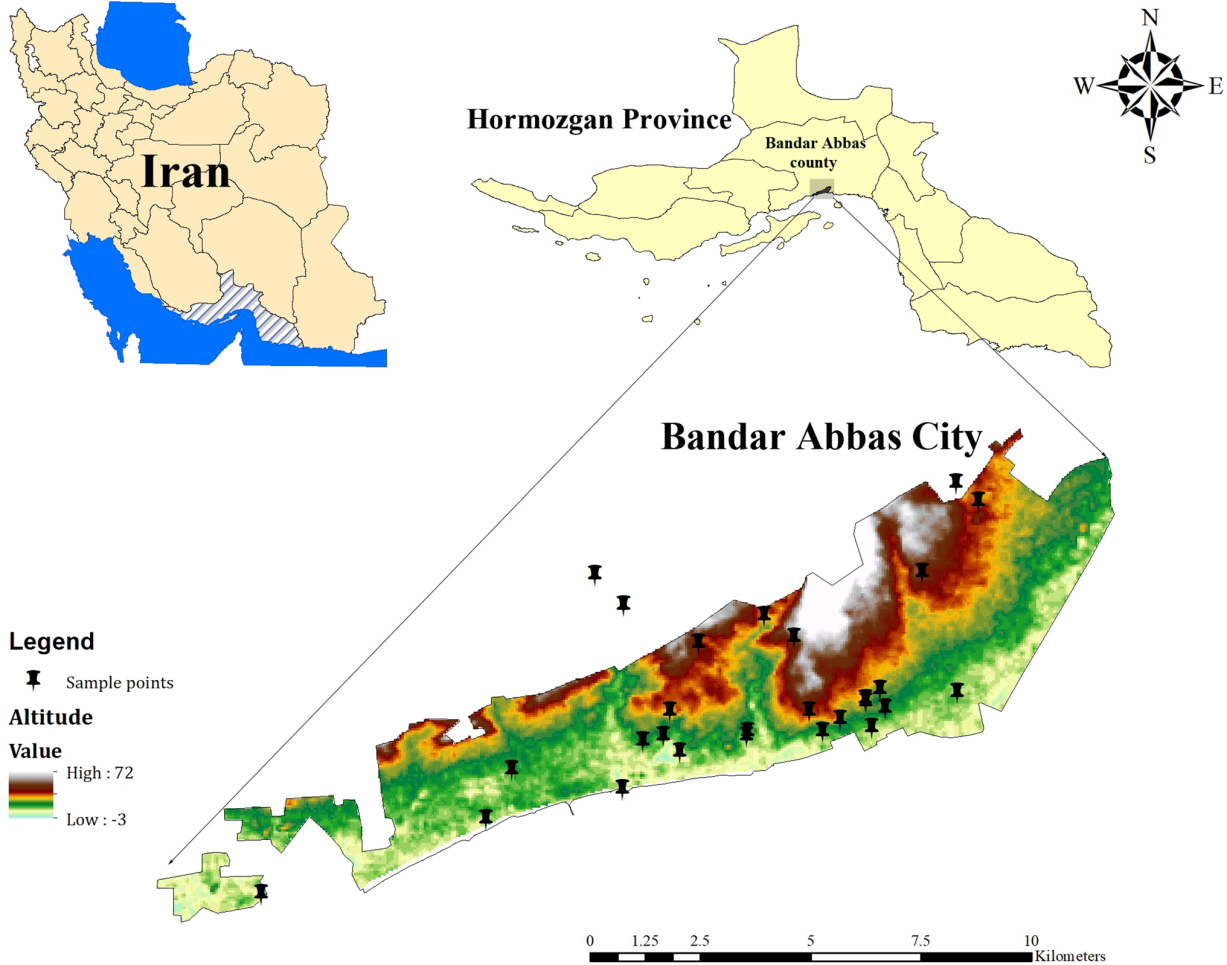
GIS Mapping of sampling sites of Bandar Abbas for Free-living amoeba

Water samples were collected from different sources such as stagnant water (3 samples), hospital water coolers (8 samples), fountains and squares (5 samples), drinking water from Hospitals (9 samples), university dormitories (13 samples), public swimming pools (42 samples), nearby hot water springs and recreational water (3 samples) in November 2019 to January 2020. A total of 83 water samples collected. About 1000 ml of water filtered through cellulose acetate filter membranes with 0.45µ pore diameter. All collected samples from 42 sites transferred to the laboratory of Bandar Abbas, Faculty of Health, during 24 h, and Physico-chemical properties of water samples were recorded. Figure 1 shows a geographic information system (*GIS*) of sampling sites of study.

#### Isolation and identification of *Acanthamoeba*

About 1000 ml water samples collected from each sampling site and were passed through a cellulose acetate filter paper, pore size diameter of 0.45µ, by a vacuum pump. The water samples, which contained visible particles, first passed through the gauze and then was filtered by the device.

The filter paper was then cultured upside down in sterile conditions on a 1.5% non-nutrient agar culture medium (NNA) enriched by a layer of Gram-negative *Escherichia coli* bacteria and incubated for two days at 37 °C. To prevent the culture medium from drying out, around the plates were sealed and tightly wrapped by parafilm during the culture or amoeba detection on microscope. After 48–72 h, the examination of plates by reverse-phase microscope continued for one month each day to detect growth and proliferation of amoeba.

#### DNA extraction

The PBS added to surface of culture plates and washed thoroughly and gently to harvest the *Acanthamoeba* from the surface of the culture medium using a sterile scraper. The collected organism kept in microtubes containing PBS, pH 7.2. Sample centrifuged 5 min in 2000 rpm to remove agar and excess materials.

The DNA extraction process was performed using the DynaBio™ Blood/Tissue DNA Extraction Mini Kit by Takapouzist company (Tehran, Iran). The DNA yield assessed by Nanodrop to estimate concentration of extracted genome.

#### PCR analysis

The PCR reaction was performed using JDP1-JDP2 primer pair for *Acanthamoeba* that can detect the genus of organism (genus-specific primer) that gives 500 bp amplicon. Also, it can identify the particular genotypes of this amoeba. To date, 20 genotypes of Acanthamoeba are known [12]. Primer pair includes the forward primer JDP1 (5-GGCCCAGATCGTTTACCGTGAA) and the reverse primer JDP2 (5-TCTCACAAGCTGCTAGGGAGTCA).

Each reaction was carried out in a final volume of 25 μl containing 1× PCR buffer, 1U Taq polymerase, 1.5 mM MgCl_2_, 200 μM of dNTPs, 10 pmol of each primer (TAG; Copenhagen A/S, Denmark) and 6 ml of the extracted DNA. Amplification parameters were performed in a DNA thermal cycler (Bio-Rad, USA). Electrophoresis of PCR products were done using 1.5% agarose gel stained with Gel Red, and amplicons were visualized under UV light. *Acanthamoeba* T4 genotype and distilled water implemented as positive and negative control, respectively.

#### Phylogenetic analysis

Each purified PCR products from the water samples and reference strains were analyzed for sequencing in both directions (Bioneer, Daejeon, South Korea). The Neighbor-Joining method was performed using the phylogenetic program MEGA version 10 [13] and verified by the maximum likelihood method with 1000 bootstrap replications [14]. The phylogenetic tree was rooted using *Naegleria americana*.

#### Nucleotide sequence accession number

The GenBank accession numbers of an investigated isolates of *Acanthamoeba* sp. determined in this study are MT645313, MT862472- MT862477, and MT863327.

### Results

In this study, 83 water samples from different water resources were collected in Bandar Abbas city. Totally nine samples of hospital water pipes, eight samples of hospital water coolers (water dispensers) that located for referral people and patient companions, 13 samples of water pipes of student dormitories, five samples of water fountains and city squares, 42 samples of swimming pools, three samples of hot springs and three samples of stagnant water were analyzed.

**Fig. 2:**
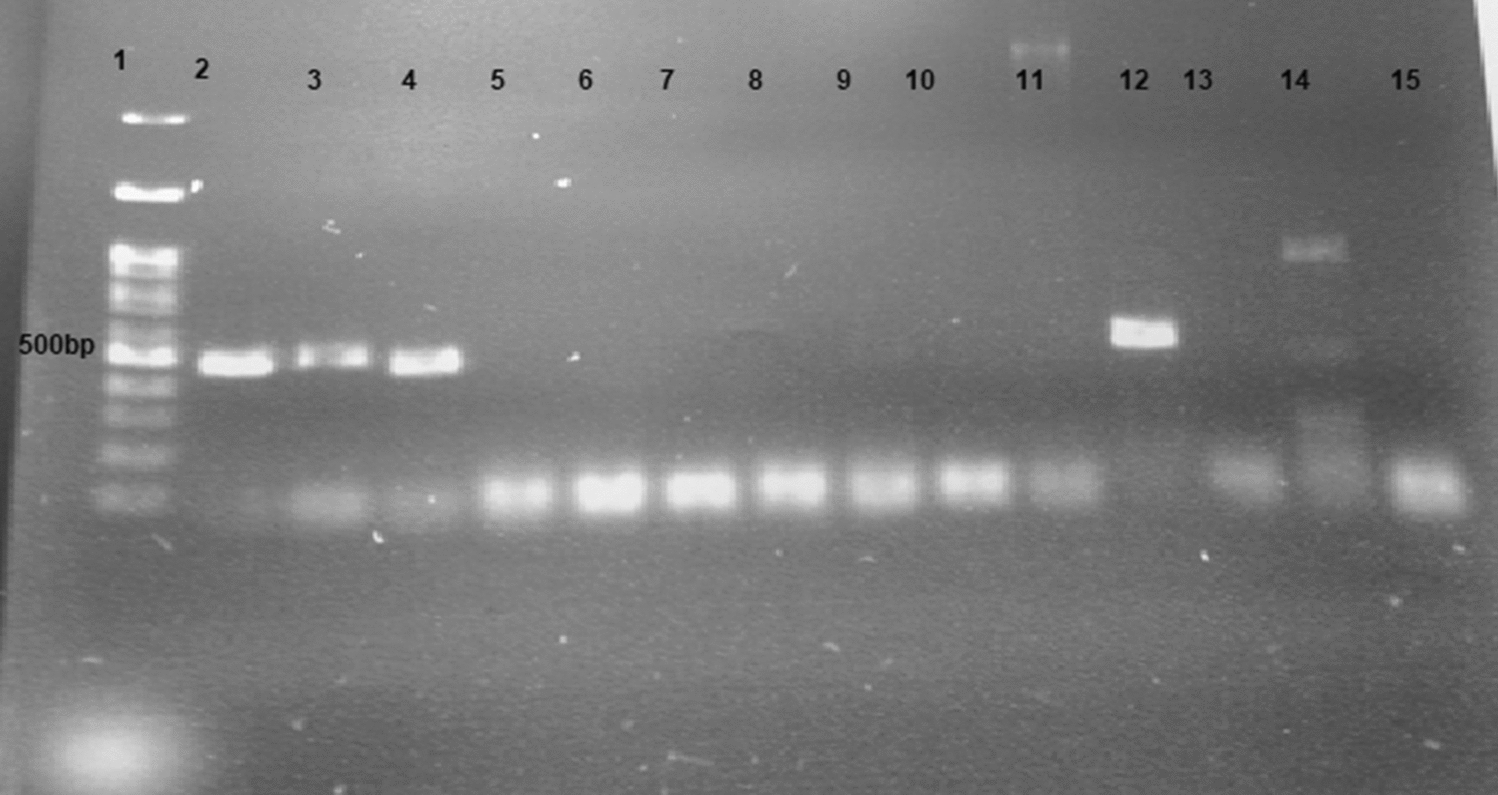
The PCR analysis of isolated amoeba recovered from water samples of Bandar Abbas. To confirm the presence of *Acanthamoeba*, DNA extracted from grown amoeba on NNA, and reaction done. As noted in material and method, the PCR produced 500 bp amplicons. Lane 1: marker: Molecular weight marker (100 bp), Lane 2: Positive Control, Lanes 3–14: Water sample, Lane 15: Negative Control

Water samples were immediately transferred to the laboratory. Using the culture method, 31 cases (37.3%) of free-living amoeba were detected. Five positive samples of *Acanthamoeba* was identified by culture methods (6%) and 8 cases (9.6%) by molecular methods (Fig. 2). The results of the number of positive samples by PCR summarized in Table 1.

**Table1:** Results of the number of positive samples recovered from the water resources by PCR

Source	Positive samples by PCR	Genotypes	GenBank accession number
Pools	3	T4, T15	MT862472, MT862474, MT863327
Fountain and squares	2	T4	MT862473, MT862475
Dormitories	2	T4	MT862476, MT862477
Tap water of hospital	1	T4	MT645313

Additional file 1: Figure S1 shows the typical cysts of *Acanthamoeba* isolated on non-nutrient agar (NNA).

#### Sequencing and genotype identification

Homology analyses of the PCR products were done using BLAST (Basic Local Alignment Search Tool) software from the NCBI. We entered the genes into the Mega-x software environment and performed the alignment, then repeated the phylogenetic tree with the Neighbor-Joining method and the bootstrap test 1000 times and draw a distance of 0.5 (Additional file 2: Figure S2).

In this study, after determining the sequence of nucleotides, two genotypes were presented as *Acanthamoeba*: Seven cases of T4 (87.5%) and one case of T15 (12.5%).

### Discussion

Our study is the first investigation that carried out in Bandar Abbas with culture and molecular methods. The results of our study showed that frequency of free-living amoebae in different water resources of Bandar Abbas city is considerable (37.3%). The study revealed that in culture method, 6% of 83 samples were positive for *Acanthamoeba*, while PCR detected 9.6%, which indicates the higher sensitivity of the molecular method in the diagnosis of *Acanthamoeba*. Studies indicate that PCR can be more sensitive and effective method in diagnosis of *Acanthamoeba* and can eliminate the need for skilled microscopist, but no single method is suggested [15–17].

In our study, some negative cases in culture method showed positive band in PCR test. Microscopic diagnosis of *Acanthamoeba* mostly rely on detecting polygonal or star shape cyst of amoeba. It may exist few cysts in some cultures that we couldn’t realize or have been misdiagnosed as artifacts.

Based on the phylogenic tree of the 18 SrRNA gene, *Acanthamoeba* T4 was the primary genotype detected in water samples from all the studied area, and phylogenic analysis showed valuable sensitivity and specificity for differentiation between each genotype. In previous studies, the T4 genotype was reported to be isolated from samples such as surface waters, drinking water, natural thermal water, swimming pools, hospital water, recreational water areas, and also soil and dust sources [18–20]. Also, our findings showed that positive cases of pools were mixes of two genotypes (T4, T15), but other positive cases were T4. Therefore, T4 considered as worldwide and isolated from clinical and environmental samples [21, 22].

El Wahab in Egypt collected 80 samples of water supplies and proved that the dominant genotype of *Acanthamoeba* is T4; this is in accordance with our results [23].

Magnet et al. reported that 94.6% of samples were positive for *Acanthamoeba* sp. by PCR, and most of them belonged to the T4 genotype, which was in line with our study [24].

Another study conducted in Italy on samples from different water matrices, *Acanthamoeba* T4 was the most common genotype detected in 13/18 isolates (72.2%), while T15 genotype was observed only in samples from Apulia and Basilicata [18].

Golestani in Kashan, Pezeshki in Zanjan, Fraji in Lorestan showed that frequency of *Acanthamoeba*is between 30 and 80% in water samples, pools tap water, and hospital pipes [23, 25, 26]. These studies are in agreement with our results, except in amount of chlorine in Lorestan study that has a significant relation with organism growth and multiplication, but in our chlorine did not affect *Acanthamoeba* viability.

In most studies, sensitivity of molecular methods in the detection of free-living amoebae were higher than culture method. We used culture and molecular methods to diagnose *Acanthamoeba* simultaneously, and high sensitivity of molecular method was confirmed.

There was a statistically significant difference between cultivation and molecular methods in identifying positive cases of *Acanthamoeba* (P > 0.001).

Polymerase chain reaction (PCR) method has been used since 1996 in identifying *Acanthamoeba*, and a recent study on its accuracy by Boggild et al. showed that it compared favorably with smear microscopy in terms of sensitivity. Still, DNA extraction should be done in very proper and precise manner, although specificity is slightly poorer [27].

The physico-chemical properties of water samples recorded in this study included pH, residual or remnant chlorine, turbidity, or external particles (if it is present should remove by gauze) and temperature that considered for each sample before filtration and cultivation.

There was no significant relationship between chlorine amount and frequency of *Acanthamoeba* by culture and molecular method (P > 0.05). Michael Storey showed that *Acanthamoeba* cysts could remain in 100 mg/l chlorine (free and combined) for 10 min [28]. In our study, *Acanthamoeba* isolated from places with 2 mg/l free chlorine.

The most positive detected cases of *Acanthamoeba* have been related to swimming pools at the temperature range of 26–30 °C. Nielsen and Naveed Ahmed Khan showed that the highest growth rate for six amoebic strains tested was close to 30–32 °C [29, 30]. Result didn't show any positive samples from geothermal hot spring water of Bandar Abbas. Some studies showed that based on the morphological characteristics of amoebae, 42% of warm spring waters of southwest of Iran are positive for *Acanthamoeba,* and others revealed that genotype T2, T4, T15 could exist in such environments [12, 31].

No significant statistical differences were observed between pH variable and frequency of *Acanthamoeba* using culture and molecular methods (p-value = 0.014) and p-value (p = 0.116) respectively. In the range of pH 7–8.3, more positive cases of *Acanthamoeba* were identified.

### Conclusion

Due to the importance of water sanitation on public health, in order to improve the quality of water resources, monitoring infectious agents like free-living amoeba, especially in swimming pools, squares and parks, water storage, and plumbing water is necessary. On the other hand, use of sensitive molecular detection along with culture method can increase the diagnosis efficacy.

## Limitations

Geothermal or hot spring waters are areas with extended regions, and we couldn't take enough samples, because obtaining representative samples of geothermal fluids requires specific sampling techniques.

## Supplementary information

**Additional file 1: Figure S1.** *Acanthamoeba* cysts (×400) on non-nutrient agar plates when observed under an inverted microscope.

**Additional file 2: Figure S2.** Phylogenetic tree for positive samples (presented with red circle), reference strain (NCBI sequences/presented with green and blue quadrangle) of water resources of Bandar Abbas. Each branch Showedthe GenBank accession number with a brief description of each sequence used. Scale bar indicates bootstrap proportion values.

## Data Availability

The GenBank accession numbers of an investigated isolates of *Acanthamoeba* sp. obtained in this study can be accessed in GenBank (GenBank: https://www.ncbi.nlm.nih.gov/) under the accession number: MT645313, MT862472- MT862477, and MT863327. All data generated or analysed during this study are included in this published article (and its additional files).
