# Supplementary figures and images for: Investigating the frequency of free-living amoeba in water resources with emphasis on Acanthamoeba in Bandar Abbas city, Hormozgan province, Iran in 2019–2020
Source: BMC Res Notes. 2020 Sep 5;13:420. doi: 10.1186/s13104-020-05267-z (PMC7487464; doi:10.1186/s13104-020-05267-z)

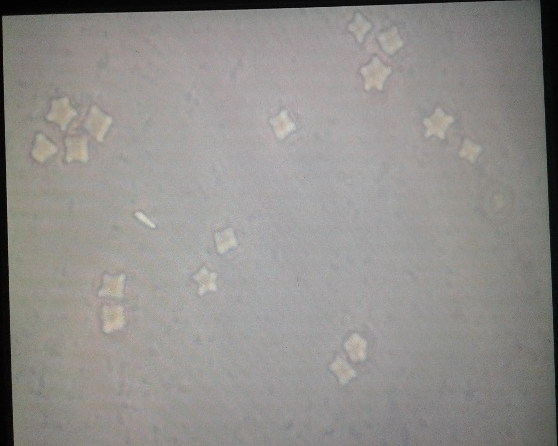

Supplement: Supplementary file 1 — Additional file 1: Figure S1. Acanthamoeba cysts (×400) on non-nutrient agar plates when observed under an inverted microscope. [file 13104_2020_5267_MOESM1_ESM.tif]

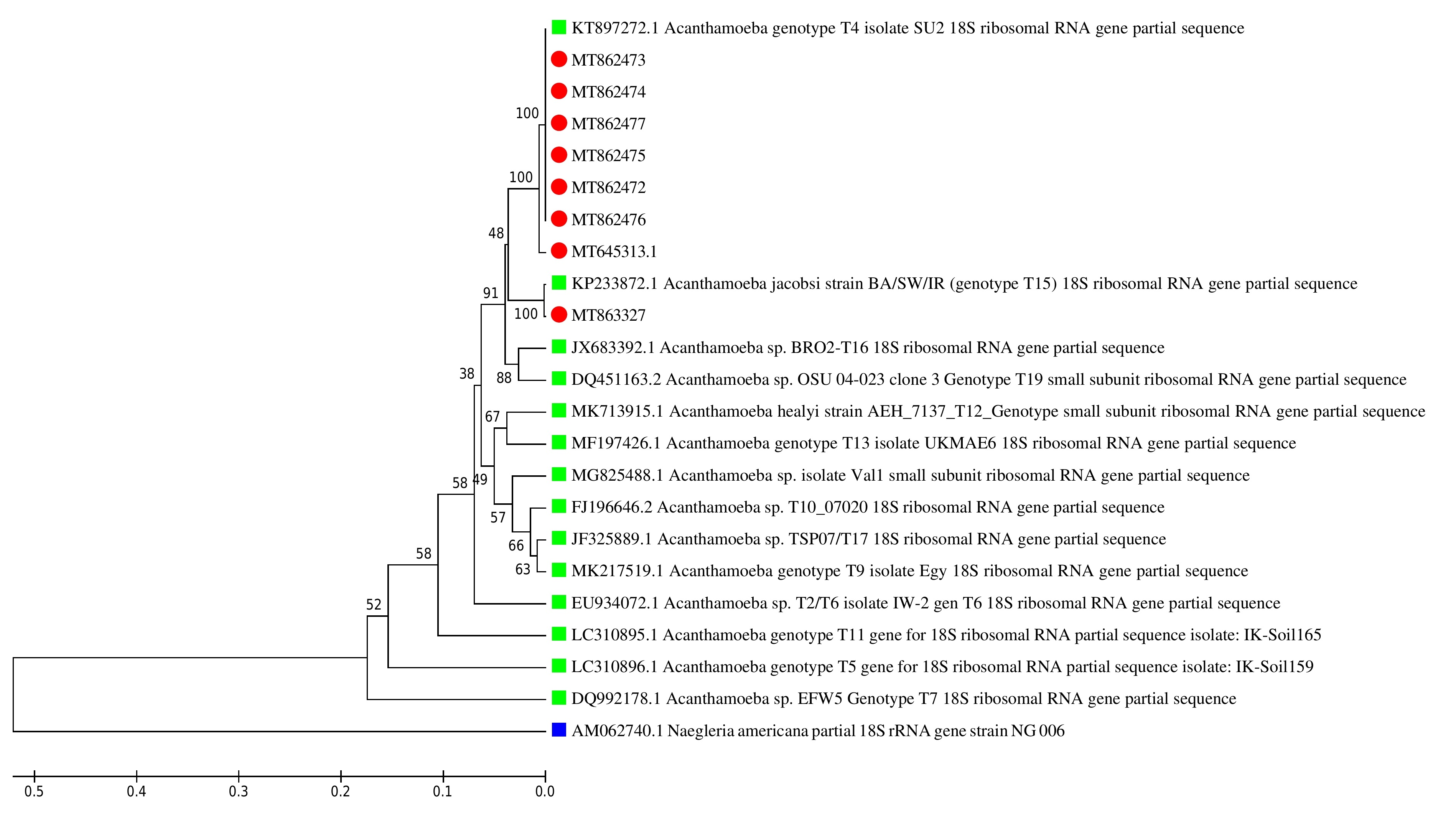

Supplement: Supplementary file 2 — Additional file 2: Figure S2. Phylogenetic tree for positive samples (presented with red circle), reference strain (NCBI sequences/presented with green and blue quadrangle) of water resources of Bandar Abbas. Each branch Showedthe GenBank accession number with a brief description of each sequence used. Scale bar indicates bootstrap proportion values. [file 13104_2020_5267_MOESM2_ESM.tif]
